# The Effect of Ankle Support on Lower Limb Kinematics During the Y-Balance Test Using Non-linear Dynamic Measures

**DOI:** 10.3389/fphys.2019.00935

**Published:** 2019-07-25

**Authors:** Herbert F. Jelinek, Kinda Khalaf, Julie Poilvet, Ahsan H. Khandoker, Lainey Heale, Luke Donnan

**Affiliations:** ^1^School of Community Health, Charles Sturt University, Albury, NSW, Australia; ^2^Department of Biomedical Engineering, Khalifa University of Science and Technology, Abu Dhabi, United Arab Emirates; ^3^Department of Biology and Computer Science, University of Poitiers, Poitiers, France

**Keywords:** tape support, ankle joint dynamics, singular value decomposition entropy, higuchi fractal dimension, Y balance test

## Abstract

**Background:** According to dynamical systems theory, an increase in movement variability leads to greater adaptability, which may be related to the number of feedforward and feedback mechanisms associated with movement and postural control. Using Higuchi dimension (HDf) to measure complexity of the signal and Singular Value Decomposition Entropy (SvdEn) to measure the number of attributes required to describe the biosignal, the purpose of this study was to determine the effect of kinesiology and strapping tape on center of pressure dynamics, myoelectric muscle activity, and joint angle during the Y balance test.

**Method:** Forty-one participants between 18 and 34 years of age completed five trials of the Y balance test without tape, with strapping tape (ST), and with kinesiology tape (KT) in a cross-sectional study. The mean and standard errors were calculated for the center of pressure, joint angles, and muscle activities with no tape, ST, and KT. The results were analyzed with a repeated measures ANOVA model (*P*_*A*_ < *0.05)* fit and followed by *Tukey post hoc* analysis from the R package with probability set at *P* < 0.05.

**Results:** SvdEn indicated significantly decreased complexity in the anterior-posterior (*p* < 0.05) and internal-external rotation (*p* < 0.001) direction of the ankle, whilst HDf for both ST and KT identified a significant increase in ankle dynamics when compared to no tape (*p* < 0.0001) in the mediolateral direction. Taping also resulted in a significant difference in gastrocnemius muscle myoelectric muscle activity between ST and KT (*p* = 0.047).

**Conclusion:** Complexity of ankle joint dynamics increased in the sagittal plane of movement with no significant changes in the possible number of physiological attributes. In contrast, the number of possible physiological attributes contributing to ankle movement was significantly lower in the frontal and transverse planes. Simply adhering tape to the skin is sufficient to influence neurological control and adaptability of movement. In addition, adaptation of ankle joint dynamics to retain postural stability during a Y Balance test is achieved differently depending on the direction of movement.

## Introduction

Postural control and balance involve coordination between proprioceptive input, individual muscle output, joints, and limbs to provide stability. Whole body biomechanical and sensory-motor feedforward and feedback mechanisms play a role in retaining postural stability. Postural stability has been investigated using both static and dynamic balance tests (Prieto et al., 1993). Taping of the lower limb including ankle or knee are common methods to improve stability, although strapping tape provides rigid support, whilst it has been suggested that kinesiology tape leads to improved proprioception (Capobianco and Van Den Dries, 2013). Dynamic movement and postural stability requires sensory, motor, and central nervous system integration (Riemann and Lephart, 2002). Kinematic and kinetic mechanisms are involved in frontal, sagittal, and transverse plane body movement (Quatman et al., 2010). Observing ankle and knee kinematics and kinetics during a dynamic postural control task such as the Star Excursion Balance Test (SEBT) or Y Balance Test (YBT) using a motion-analysis capture system provides the opportunity to evaluate dynamic, nonlinear patterns using fractal and entropy measures and may lead to a better understanding of lower limb taping efficacy (Doherty et al., 2015). Traditional laboratory measures of postural stability in static positions is not sensitive enough to detect small multiphase kinematic and kinetic changes associated with postural stability nor can these test results be extrapolated to dynamic movement stability (Hrysomallis et al., 2007; Mckeon and Hertel, 2008). Both the SEBT and YBT are established dynamic postural functional tests. The SEBT consists of a series of maximal single-leg extensions using the—non-stance leg along eight designated lines arranged in a grid on the ground that extends from a center point and are 45°From one another (Gribble et al., 2012). The YBT differs to the SEBT that it only requires extension of the non-stance leg in three directions, 120 degrees apart from the center (Shaffer et al., 2013) in the anterior, posterior-medial, and posterolateral reach direction (Fullam et al., 2014). Ankle and knee joint angles, as well as muscle myoelectric activity are the main physiological parameters that provide information on ankle stability (Kodesh and Dar, 2015; Vigotsky et al., 2018). The center of pressure (CoP) is also commonly used during balance tests to assess postural stability, and can be combined with joint kinematics and myoelectric muscle activity (Doherty et al., 2015). Surface electromyography (sEMG) characteristics correspond to a set of coordinated contractions of different muscles that collaborate to restore balance and retain postural stability (Vieira et al., 2011).

Rigid strapping tape (ST) is commonly used to stabilize the ankle joint and improve balance by limiting range of motion at selected joints. It is also a means to prevent lateral ankle sprains, especially when performing sporting activities (Griffin and Bernhardt, 2006). An alternate taping option when attempting to improve stability is the use of fascial taping techniques, whereby tape is applied to the skin using decompression technique. Upon application, differential surface tension is induced, lifting the skin and allowing fluids to flow more freely (Capobianco and Van Den Dries, 2013). If applied correctly, this is believed to impact the activity of both mechanoreceptors and afferent nociceptors (Alexander et al., 2008). While the evidence supporting the skin taping approach is limited to case reports by those that proposed the particular techniques (Chen et al., 2013), the approach is in complete contrast to traditional taping methods whereby tension is applied to the tape upon application, and range of motion is restricted (Capobianco and Van Den Dries, 2013). It is recommended that kinesiology tape is applied with little to no tension while the target tissue (skin above the muscle or joint) is in a stretched position (Capobianco and Van Den Dries, 2013).

Using linear measures, studies have already shown better results in improving dynamic balance and ankle stability outcomes on the SEBT using KT as compared to ST in patients with first degree ankle sprain (Mervat et al., 2016), and increased dynamic postural control in females when reaching in the posterior-medial and medial directions during the SEBT (Nakajima and Baldridge, 2013). In this study it is hypothesized that non-linear analysis of relevant associated kinetic and kinetic parameters may provide a better understanding of the effectiveness of ST vs. KT in postural control dynamics.

The Fast Fourier Transform (FFT) is a common linear analysis method applied for characterizing sEMG activity or amplitude changes. However, it has inherent limitations since sEMG signals are irregular, complex and non-stationary (Raez et al., 2006). Similarly, CoP displays chaotic dynamics that fluctuate over time with increasingly smaller amplitude variations at shorter time scales, suggesting a self-similar or scale-invariant process (Müller et al., 2017). These complex physiological characteristics, therefore, suggest that non-linear dynamic approaches, such as fractal analyses and entropy, may provide practical alternatives for analyzing these complex, non-linear and non-stationary sEMG, kinematic and kinetic (joint angle and CoP) measures during a Y Balance Test (Peng et al., 1992; West, 2014).

The Higuchi fractal dimension (HDf) quantifies the complexity of the scaling variability associated with biosignals, such as CoP, muscle contraction and muscle bundle recruitment dynamics (Higuchi, 1988). It has previously been successfully applied to clinical neurophysiology (Kesić and Spasić, 2016), sEMG analysis (Truong Quang Dang et al., 2012), as well as the analyses of CoP during postural control (Doyle et al., 2004). However, HDf has not been applied to study sEMG dynamics associated with postural adjustment during balance tests. In particular, there are no studies to date that use fractal analysis, such as HDf, to investigate the effectiveness of taping to improve postural stability.

Entropy is also a characteristic of non-linear, complex systems, and indicates the extent of regularity in a system, that is, a certain signal characteristic is likely to occur again (Lebon et al., 2008). Several entropy-based features have been proposed to determine the complexity of biosignals, including, Sample Entropy, Approximate Entropy, Rényi entropy and Singular Value Decomposition Entropy (SvdEn) (Lake et al., 2002; Zhang et al., 2012; Caraiani, 2014; Cornforth et al., 2014). SvdEn is an extension of principle component decomposition analysis, defined in this work as the number of attributes required to characterize a biosignal (sEMG, CoP joint angle dynamics). Biosignals are represented in matrix form in order to extract singular values from the matrix representation of the signals, and entropy is determined from the data associated with each singular value. In this case entropy is the aggregate measure of the information content for the data associated with the selected singular values describing the sources of the biosignals (Caraiani, 2014).

Entropy and fractal analyses have both proven useful in analyzing postural adjustment during balance testing, such as the SEBT or YBT (Doherty et al., 2015). SvdEn has the advantage of enabling the analysis of very short and non-stationary data sets (Li et al., 2008) and has previously been applied to describe the CoP associated with postural sway (Sabatini, 2000). However, entropy and fractal measures have not been applied to measure kinematic and kinetic responses during a dynamic postural balance test. These measures may be more suitable to identify non-linear, non-stationary effects of taping on postural stability and associated hierarchical sensorimotor adjustments along the neuroaxis during movement (Von Laßberg et al., 2017).

This current study investigated the use of SvdEn and HDf to determine kinematic and kinetic adjustments during the YBT as these measures provide additional non-linear information and are robust for short time series and discern whether applying either kinesiology tape or strapping tape influenced muscle activity, joint angle dynamics, or CoP as compared to no tape.

## Methods

### Participants

Forty-one male (*n* = 21) and female (*n* = 20) participants (mean ± sd; Age: 22.0 ± 3.0 years, Height: 1.74 ± 0.1 m, Weight: 75.9 ± 12.8 kg) aged 18–34 years completed this study. Based on a power analysis with Power set at 0.8, large effect size and *p* ≤ 0.05 (G^*^Power 3.1.9.4 for Windows), the sample size required is 34 participants (17 in each group) (Faul et al., 2007). Participants were free of musculoskeletal, cardiovascular, or neurological injury. Pregnant females were excluded from the study due to the confounding effects of the relaxin hormone on the joint range of motion, causing inherent changes in weight distribution and therefore balance. The Charles Sturt University Human Ethics Committee approved the study, and all participants provided written consent having been informed about the research process and aims in accordance with the Declaration of Helsinki.

### Y Balance Test

**Figure 1:**
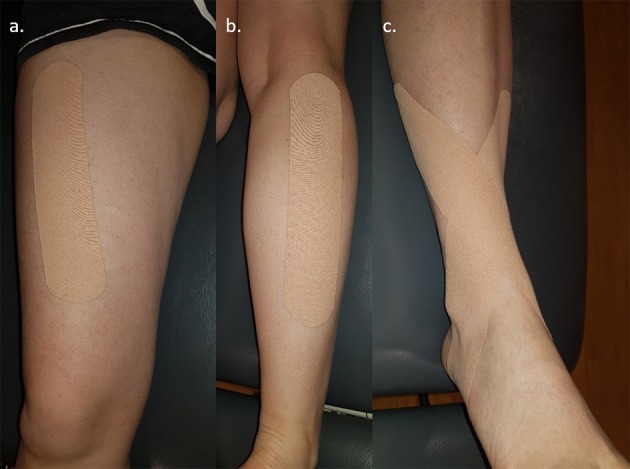
Location of tape application on dominant leg: **(a)** anterior thigh—rectus femoris; **(b)** posterior leg—gastrocnemius; **(c)** ankle—anti-inversion ankle strap.

The Y Balance test (YBT) requires the participant to balance on their dominant leg, and reach with their non-dominant leg as far as possible in the anterior, posterolateral, and posteromedial directions (as shown in Supplementary Material—Item 1). In this study, all participants completed the YBT with no tape, ST (5 cm, Leauko sports tape), and KT (5 cm, Kinesio Tex® Tape). A single strap was applied to the muscle belly of both the rectus femoris (Figure 1a) and the gastrocnemius (Figure 1b), in accordance with published fascial movement taping techniques (Capobianco and Van Den Dries, 2013). A single anti-inversion strap was also applied to the foot and ankle, starting on the inside of the lower tibia, extending over the lateral malleolus, crossing under the arch of the foot, and over the medial malleolus, to finish at the lateral border of the lower tibia overlying the fibularis muscle (Figure 1c) (Kodesh and Dar, 2015). The rectus femoris and gastrocnemius muscles were assessed as they control knee and ankle, respectively, as the body is lowered during the maximal reaches of the YBT. This control is achieved through eccentric contractions, whereby the muscles elongate under tension as a means to slow or limit joint movement (Visnes and Bahr, 2007; Maeo et al., 2018). The ankle-foot strap was also applied as a means to limit the side to side movement of the ankle during full weight bearing, given the potential for side to side movement of the foot to impact lower limb mechanics during a squat (Lee et al., 2015). All straps were applied without tension, and the rectus femoris and gastrocnemius straps were applied while the muscle was undergoing stretch (Capobianco and Van Den Dries, 2013). While the strapping methods used were congruent with recommended fascial movement taping techniques, a strapping tape was also applied in this fashion to normalize and eliminate any bias due to tape application. All taping was completed by an experienced clinician trained in fascial movement taping techniques.

Performance of the YBT under each taping condition was repeated five times, and the average of five trials was calculated. The order of taping variables was randomized, and testing was undertaken 3 days apart to limit any learned effect. On a single testing day, participants were tested once with tape, and once without tape. The first day, participants were randomly assigned to either start by comparing the KT to no tape, or perform the YBT using ST compared to no tape. After a 3-day washout period, the participant returned to repeat the testing procedure with the alternate taping method. Prior to testing, participants completed a 5 min self-paced treadmill warm-up, and a further 5 min of treadmill activity between taping and no tape testing, to reduce the learned effect of the YBT, maintain muscle readiness, and reduce the risk of injury (Yapicioglu et al., 2013).

### Data Processing

**Figure 2:**
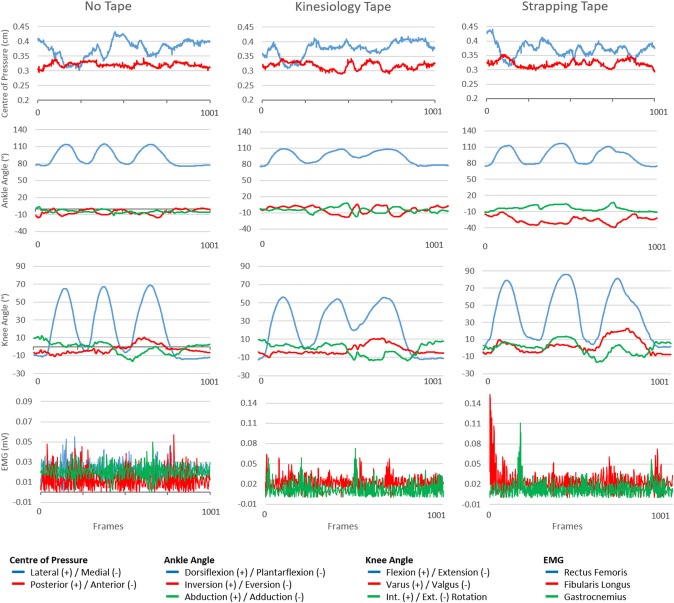
Graphing of center of pressure, ankle angle, knee angle, and electromyography values across three taping variables (no tape, kinesiology tape, strapping tape) over a normalized 1001 frame time series.

Initial data processing was completed using Visual 3D (Version 6, C-Motion, Germantown, MD), where joint angle and ground reaction force data underwent fourth-order Butterworth low-pass filter with cut-off frequencies at 18 and 50 Hz, respectively. A Cartesian local coordinate system defined the y-axis as lateromedial, x-axis as posterior-anterior, and z-axis as external-internal rotation (Supplementary Material—Item 1). Intersegmental joint angles were calculated at the ankle and knee joints. Dry surface EMG electrodes containing a pre-amplification attachment (1,000 Hz, Trigno™ 16 channel wireless EMG system, Delsys Inc, USA) were positioned in accordance with SENIAM guidelines to obtain accurate muscle myoelectric activity readings. Skin preparation involved shaving of the skin at the application site to remove both hair and loose epidermal cells, and wiping with an alcohol impregnated wipe (Hermens et al., 1999). All sensors were tested during the initial calibration phase to ensure a suitable signal was being obtained. Raw sEMG data was rectified, and filtered with a high band pass filter cut off frequency set at 20 Hz to reduce noise interference (Wang et al., 2013). The low band pass filter cut-off frequency was set at 450 Hz, followed by a Chebyshev filter, to obtain a linear envelope of the EMG. Each individual time series was normalized to 1001 frames to allow direct comparison between all trials (Figure 2). Joint angles and ground reaction forces while performing the YBT were recorded with an eight-camera Vicon 3-D motion analysis system (100 Hz, model, Vicon, Oxford Metrics, UK) and single AMTI force plate (1,000 Hz, BP600900-2K, AMTI, USA). All data was time synchronized and recorded with Vicon Nexus (v1, Oxford Metrics, UK).

### Programming

All complexity measures were calculated using a Python script based on the complexity.py script available through the Neurokit package (https://pypi.org/project/neurokit/) for purposes of the current analysis. sEMG signals were filtered with a script downloaded from Scientifically Sound (https://scientificallysound.org/2016/08/22/python-analysing-emg-signals-part-4/) prior to determining HDf and SvdEn.

### Singular Value Decomposition Entropy

SvdEn is optimal for describing very short and non-stationary signals. The amount of information within a signal, is related to the signal's attributes and identified as singular values. Greater information content is associated with more attributes and a higher SvdEn. In our proposed model of SvdEn, a high CoP SvdEn translates to more physiological attributes being associated with postural sway, more complex joint angle dynamics, and more muscle bundle switching.

The SVD entropy is defined as:

$$\begin{array}{l} H_{SVD}=\;-\;\overset{M}{\underset{i=1}{\sum }}{\bar{\sigma }}_{l}\;{\text{log}}_{2}{\bar{\sigma }}_{i} \end{array}$$

where *M* is the number of singular values and σ*1,…*, σ_*M*_ are normalized singular values. The equation of SvdEn requires a delay and an embedding dimension (Li et al., 2008). For the current study the delay was defined as one and the embedding dimension equal to two.

### Higuchi Fractal Dimension

The Higuchi fractal dimension (HDf) quantifies the degree of fluctuation of the time series. The HDf is defined as:

$$\begin{array}{l} L\left(k\right)=\;\frac{1}{k}\;\times \overset{k}{\underset{m=1}{\sum }}L\left(m,k\right) \end{array}$$

where *k* is the discrete time interval between points, *m* represents the initial time value, and *L(k)* is the length of the curve for the time interval *k*. The maximal *k* was set at eight (Kesić and Spasić, 2016; Čukić et al., 2017). The Higuchi dimension is considered the gold standard for estimating dimensional complexity of a time series due its robustness against noise and suitability for short recordings (Spasic et al., 2005). Higher HDf values indicate more complex movement variability, and greater ankle, and muscle dynamics.

### Statistics

The mean and standard error (SE) were calculated for the CoP, joint angles and muscle activities with no tape, ST and KT. To determine the relevance of the results, a repeated measures ANOVA model (*P*_*A*_ < *0.05)* was applied followed by the *Tukey post hoc* method from the R package for comparing a family of three estimates (no Tape, Strapping Tape, and Kinesiology Tape), to allow for *P*-value adjustment.

## Results

To determine the effect of taping using HDf data was first analyzed using a repeated measures ANOVA followed by the Tukey *post hoc* test to adjust the obtained *p*-values and allow for multiple comparisons.

### Effect of Taping—SvdEn

No significant effects of taping were observed for the CoP using a repeated measures ANOVA (*P*_*A*_ > 0.05) and Tukey *post hoc* analysis in the anterior-posterior (*P*_*A*_ = 0.40) and mediolateral directions (*P*_*A*_ = 0.28) (Table 1), despite the increased SvdEn.

**Table 1:** SvdEn results of CoP and taping during YBT.

	***NT***	***ST***	***KT***
CoP*			
AP	0.021 ± 0.003	0.024 ± 0.003	0.026 ± 0.003
ML	0.052 ± 0.004	0.059 ± 0.004	0.058 ± 0.004

* *CoP-Center of Pressure, NT-no Tape, ST-strapping tape, KT-kinesiology tape, AP-anterior-posterior, ML-mediolateral, mean ± SE*.

Similarly, repeated measures ANOVA results at the knee joint were not significant in the anterior-posterior (*P*_*A*_ = 0.95), mediolateral (*P*_*A*_ = 0.25), and internal-external rotation (*P*_*A*_ = 0.61) directions during the YBT (Table 2) and no *post hoc* analysis to correct for multiple elements was performed.

**Table 2:** SvdEn results of knee and ankle angle changes during the YBT.

	**Knee Joint Angle**			**Ankle Joint Angle**		
	NT*	**ST**	**KT**	**NT**	**ST**	**KT**
AP	0.041 ± 0.002	0.041 ± 0.002	0.042 ± 0.002	0.0095 ± 0.001	0.0045 ± 0.001	0.0043 ± 0.001
ML	0.129 ± 0.015	0.146 ± 0.015	0.132 ± 0.015	0.27 ± 0.02	0.27 ± 0.02	0.30 ± 0.02
SI	0.083 ± 0.005	0.078 ± 0.005	0.078 ± 0.005	0.030 ± 0.002	0.007 ± 0.002	0.006 ± 0.002

* *NT-no Tape, ST-strapping tape, KT-kinesiology tape, AP-anterior-posterior, ML-mediolateral, SI-internal-external rotation, mean ± SE*.

However, the ANOVA and *post hoc* analysis, for the ankle joint dynamics during the YBT, found a significant decrease in SvdEn in the anterior-posterior direction (*F*_(2,114)_ = 7.12; *P*_*A*_ = 0.0015) as well as the internal-external rotation (*F*_(2,114)_ = 70.56, *P*_*A*_ = 0.0001) in association with taping. The anterior-posterior direction joint angle dynamics were significantly lower for ST as compared to NT (*P* = 0.006), and KT as compared to NT (*P* = 0.004). SvdEn for the internal-external rotation of the ankle joint movement was also significantly lower when comparing taped (KT & ST) and non-taped conditions (*P* = 0.0*001)* shown in Table 2. No significant difference was found for KT vs. ST in any direction.

The ANOVA showed a strong trend related to the effect of lower limb taping on the gastrocnemius muscle myoelectric activity with SvdEn analysis (*F*_(2,117)_ = 2.96, *P*_*A*_ = 0.057). KT led to a reduction, whereas ST led to a slight increase in SvdEn as compared to NT. However, a significant difference was seen between ST and KT (*P* = 0.047) (Table 3).

**Table 3:** SvdEn for gastrocnemius, fibularis longus, and rectus femoris muscles for taping during the YBT.

	NT*	**ST**	**KT**
Gastrocnemius	0.903 ± 0.005	0.909 ± 0.005	0.893 ± 0.005
Fibularis longus	0.905 ± 0.005	0.91 ± 0.005	0.899 ± 0.005
Rectus femoris	0.912 ± 0.006	0.911 ± 0.006	0.897 ± 0.006

* *NT-no Tape, ST-strapping tape, KT-kinesiology tape, mean ± SE*.

Kinesiology taping also led to lower SvdEn for the rectus femoris and the fibularis longus muscles as compared to ST but were not significant (Table 3).

### Effect of Taping—HDf

Repeated measures ANOVA analysis of the CoP did not reveal any significant changes in variability for the anterior-posterior (*P*_*A*_ = 0.64) and mediolateral directions (*P*_*A*_ = 0.78) (Table 4).

**Table 4:** HDf for CoP during the YBT.

	**NT**	**ST**	**KT**
CoP*			
AP	1.51 ± 0.019	1.52 ± 0.019	1.52 ± 0.019
ML	1.56 ± 0.03	1.56 ± 0.03	1.57 ± 0.03

* *CoP-center of pressure, AP-anterior-lateral, ML- mediolateral, NT-no Tape, ST-strapping tape, KT-kinesiology tape, mean ± SE*.

Changes in the knee angle associated with taping in the anterior-posterior (*P*_*A*_ = 0.6), mediolateral (*P*_*A*_ = 0.13), and internal-external rotation (*P*_*A*_ = 0.32) directions during the YBT were also not significant (Table 5). However, ankle angle dynamics during the YBT revealed a significant ANOVA (*F*_(2,114)_ = 18.5, *P*_*A*_ = 0.0001) with a significantly higher HDf for both KT and ST vs. NT with *P* < 0.0001 in the mediolateral direction only (Table 5).

**Table 5:** HDf mean of the ankle, knee angle for taping during YBT.

	**Knee Joint Angles**			**Ankle Joint Angles**		
	NT*	**ST**	**KT**	**NT**	**ST**	**KT**
AP	1.009 ± 0.001	1.009 ± 0.001	1.007 ± 0.001	1.10 ± 0.01	1.13 ± 0.01	1.11 ± 0.01
ML	1.1 ± 0.01	1.1 ± 0.01	1.09 ± 0.01	1.49 ± 0.034	1.69 ± 0.034	1.68 ± 0.034
SI	1.08 ± 0.008	1.07 ± 0.008	1.08 ± 0.008	1.56 ± 0.03	1.56 ± 0.03	1.57 ± 0.03

* *AP-anterior-lateral, ML- mediolateral, SI- internal-external rotation, NT-no tape, ST-strapping tape, KT-kinesiology tape, mean ± SE*.

HDf analysis of muscle myoelectric activity during the YBT showed a strong trend for the gastrocnemius muscle when using KT or ST when applying the repeated ANOVA (*F*_2,117_ = *2.86, P*_*A*_ = 0.06) compared to no tape, similar to the findings with SvdEn (Table 6), but was not significant following the Tukey *post hoc* test to allow for multiple comparisons (*P* = 0.062).

**Table 6:** HDf of gastrocnemius, fibularis longus, and rectus femoris muscles.

	NT*	**ST**	**KT**
Gastrocnemius	1.995 ± 0.007	2.0 ± 0.007	1.978 ± 0.007
Fibularis longus	1.988 ± 0.008	1.995 ± 0.008	1.981 ± 0.008
Rectus femoris	1.995 ± 0.01	1.995 ± 0.01	1.97 ± 0.01

* *NT-no Tape, ST-strapping tape, KT-kinesiology tape, AP-anterior-posterior, ML-mediolateral, mean ± SE*.

## Discussion

Postural control is a complex continuous process regardless of whether static stance or dynamic movement is taking place. Many physiological signals exhibit complex non-linear fractal scaling and indicating possible common feedforward and feedback control mechanism on different time scales (Ivanov et al., 2009). Hierarchical processing associated with postural stability, from the spinal cord level to the cortical regions, functions at different time scales and amplitudes leading to muscle myoelectric activity and a final common output, such as change in joint dynamics. Physiological signals, including muscle myoelectric activity, changes in joint angle dynamics, or center of pressure fluctuations, can typically be described in terms of their regularity (amount of information they contain), as well as their complexity. In this study, sEMG signals of the gastrocnemius, fibularis longus and rectus femoris, ankle and knee joint angle dynamics, and CoP were analyzed using non-linear dynamic methods that allow for the inherent non-linearity and non-stationarity observed in these parameters during the YBT (Diedrichsen et al., 2010). Fractal measures have been applied previously to assess posture and locomotion in humans and animals. Multi-scale dynamics have demonstrated robust scale-invariant and non-linear features for human in forearm motion during activities of daily living and suggest that these measures have sufficient sensitivity to investigate short movement patterns associated with a standard balance test as undertaken in the current study (Hu et al., 2004). Within the scope of movement variability, Perakakis et al. have pointed out that the emotional milieu may play a crucial role in approach and avoidance behavior. These authors measured postural sway while subjects stood on a force platform viewing pictures from three affective categories: pleasant, unpleasant, and neutral (Perakakis et al., 2012). Our study was unlikely to be influenced by emotional factors as it was located in the movement laboratory and participants were familiar with the space and the principle investigators.

According to previous studies on postural sway whilst standing, a loss of CoP complexity, measured with detrended fluctuation analysis, is due to the reduction in postural control system functionality (Zhou et al., 2013). Conversely, a high entropy, and thus high CoP irregularity, has been correlated with more efficient balance control using sample entropy. Higher sample entropy values may reflect a more automatic, less constrained balance control (Borg and Laxåback, 2010). A low degree of postural complexity observed in the CoP, has also been correlated with a low degree of postural control adaptation capacity in case of perturbation (Manor et al., 2010).

According to our results, the CoP did not change with taping when analyzed with SvdEn or HDf. This agrees with previous work, showing no influence of elastic taping on the CoP during static or dynamic balance tasks, although decreased postural control was reported with strapping tape (Abián-Vicén et al., 2008). The absence of CoP variation may also reflect our assessment of single leg stance during the YBT. Given that the CoP must reside within the base of support, which in this case is beneath the stance foot, the potential for significant variation is somewhat limited. These findings may have been different had the center of mass been assessed instead of the CoP.

Similarly, there was no significant difference between no tape and taping conditions, for knee angle dynamics during the YBT. Gribble et al. reported that participant fatigue created kinematic pattern changes that led to changes in knee angle during the SEBT but that taping in normal control participants made no difference in maximum reach distance during the SEBT (Gribble et al., 2012). The authors concluded that prophylactic support is designed to create a more stable ankle, but does not appear to consistently improve dynamic stability. Kodesh and Dar (2015) reported results comparing no tape with kinesiology tape for maximum leg extension in the AP, ML, and MP directions of the YBT. Their cohort consisted of participants with chronic ankle instability whilst their experimental paradigm included a fatigue protocol. The results of Kodesh and Dar (2015) indicated no difference between no tape and KT during the YBT. Our work did not include these features and concentrated on non-linear features associated with ankle and knee angle, CoP, and electromygraphic muscle activity. However, differences in findings may be also due to the shortened YBT being less physically demanding.

Proprioception at the ankle joint is one of the most important elements contributing to the control of balance. Taping is often used to support joints, but also decreases freedom of movements. According to manufacturers, KT adhesive taping is able to provide improved dynamic support and potentially enhance afferent sensory stimulation when compared to conventional strapping techniques, which improves proprioception as part of the sensorimotor neural response system and better postural control (Capobianco and Van Den Dries, 2013). In this study, taping did have a significant effect on the ankle joint dynamics.

A reduction in SvdEn identified a decrease in the number of physiological attributes, contributing to ankle movement for both KT and ST conditions in the anterior-posterior and internal-external rotation directions. Whereas, HDf, which is measure of the complexity of ankle dynamics, was shown to increase significantly for both KT and ST in the mediolateral direction. Interestingly, having not observed an increase in HDf in the anterior-posterior and internal-external rotation directions, this may imply that the changes to SvdEn were not related to movement complexity, but instead to the reduction in physiological attributes associated with the movement. This is possibly a reflection of a change in the neurological processing, or increased stability of the ankle in the anterior-posterior and internal-external rotation directions in the presence of tape.

If viewing the reduction in SvdEn from a purely anatomical perspective, the ankle strapping is applied with a view to inverting the foot. When moving into an inverted subtalar joint position, the midtarsal joint is believed to become “locked,” therefore limiting overall flexibility of the foot (Blackwood et al., 2005). The reduction in SvdEn relating to internal and external rotation may reflect a restriction in available motion in the transverse plane. While the ankle joint does allow some transverse plane motion (Nester et al., 2003) in what is predominantly a sagittal plane joint (Brockett and Chapman, 2016) both inverting the subtalar joint and offering external support may have limited the number of attributes required for regular corrections via proprioceptive pathways. The inherent plantarflexion that is associated with an inverted foot (Brockett and Chapman, 2016) may have also brought about the reduction in SvdEn in the anterior-posterior direction, as the ankle joint motion may be restricted by soft tissues under tension. Furthermore, an increase in plantarflexion is also associated with a reduction in frontal plane stability (Bhaskaran et al., 2015), providing further support for an increase in HDf mediolaterally. As participants lowered their body position prior to each of the three reaches, the majority of the downward movement was achieved through sagittal plane flexion of the hip, knee and ankle. As the sagittal plane movements were all intentional, and controlled eccentrically by the gluteal, quadriceps and gastrocnemius muscles, frontal plane motion was not a primary consideration, potentially justifying a compensatory movement in the frontal plane to maintain balance. From a neurophysiological perspective we can stipulate that if HDf increases and SvdEn does not, then we have greater movement variability without an increase in the number of contributing attributes. That is, there is possible less neuronal integration required along the sensory-motor axis in either the feedback or the feedforward direction. Alternatively, an increase in SvdEn without an increase in HDf, implies that the movement retains its complexity with taping, but utilizes more attributes. This implies possibly fine tuning and greater integration accuracy (hence more attributes) along the sensory-motor axis to obtain the final output.

The significant changes observed in the ankle joint dynamics can be related to muscle myoelectric activity during the balance test, as limitations of gastrocnemius extensibility can contribute to a more everted foot position in the frontal plane. There were, however, no significant changes in the electromyographic muscle activity noted at the fibularis longus and rectus femoris, and SvdEn changes to gastrocnemius were only found to be trending. Minimal changes to muscle myoelectric activity may challenge the suggestion that KT application increases afferent activity, and that resulting joint changes reflect an effect isolated to joint motion but not the supporting structures. Alternatively, gross and intentional movements may override the potential sensory effects of facilitative taping.

Similar findings were noted at the rectus femoris, with no significant changes observed. While no previous studies that we are aware of have investigated the role of the rectus femoris in dynamic postural control during the YBT, overriding of facilitative tape effects may also exist in this case. Although the rectus femoris as knee extensor, acts concentrically, it also controls knee flexion eccentrically, and is considered the main muscle controlling weight during single leg stance. Signal regularity and complexity for the sEMG activity of the rectus femoris and fibularis longus muscles did not change due to taping in our study. This observation agrees with previous work, which reported no significant effect on response latency of the fibularis longus whilst strapping the ankle (Correia et al., 2016).

Increased gastrocnemius muscle myoelectric activity and greater ankle motion were associated with lower SvdEn and HDf using KT and slightly elevated SvdEn and HDf with ST, as compared to no tape. Significantly higher ST, as compared to KT, using SvdEn may be indicative of increased gastrocnemius muscle myoelectric activity and greater contribution of the proprioceptive and somatosensory systems in the muscle response. Decreased regularity is a likely indicator of increased torque requirement to oppose the decreased ankle flexibility, following strapping taping application, which predominates muscle regularity due to sensorimotor feedback associated with kinesiology taping (Di Giulio et al., 2009). Maximum gastrocnemius electromyographic muscle activity decreases with a decrease in knee joint angle, but is also a function of muscle contractile behavior. This can explain the lack of taping effect, as the maximum flexion in the knee angle had minimum effect on gastrocnemius flexion dynamics in our study (Merlet et al., 2018). Our findings support the use of non-linear measures to investigate short time interval dynamic data as is the case of ankle and knee angle dynamics, CoP. and electromyographic muscle activity during a short balance test.

A number of limitations need to be mentioned here relating to the use of HDf and SvdEn. When applying the Higuchi dimension to calculate fractal or non-linear properties of time signals the number of data points should be at least 15,000. Although during the SEBT more data points have been observed, it is more difficult to obtain this number with the YBT as it is a shorter test. The number of data points in related to the sampling frequency, which should not exceed 500 Hz as any faster sampling frequency would not be consistent with the physiology measured. The choice of the scaling parameter *k*_*max*_, is also important and should be set at ~40, which corresponds to 0.08 s and is equivalent to about twice the minimal reaction time recording in human subjects (Zhou et al., 1996; Müller et al., 2017). Svd is inherently a linear method and is a direct matrix inversion method that requires significant computer resources. However, Svd allows for an unbiased differentiation between signal and noise, which is an important factor in biosignals analysis. A subset of singular values and vectors then represent the signal, which provide different phase information about the time series that can be investigated as an entropy process (Leblond et al., 2010).

Sensorimotor integration and the associated movement variability depend on feedforward and feedback information pathways along the neuroaxis. Movement variability, as reflected by the biosignal complexity measured using HDf, provides a global measure of movement output. SvdEn provides insight into the possible regulatory mechanisms associated with movement complexity and measures the possible number of factors contributing to the complexity of the signal. Such complexity typically increases from a simple reflex controlled at the spinal cord level only to movement controlled at level to brainstem, subcortical, and cortical levels.

## Conclusion

Utilizing HDf and SvdEn measures in this work provided a novel perspective on ankle stabilization using different types of tape and differences in the possible mechanisms and attributes contributing to ankle joint dynamics and movement variability, in lieu of traditional single parameter measures, such as the range of motion, typically used to quantify the effectiveness of taping. A significant decrease in SvdEn was observed in the anterior-posterior direction of joint motion as well as the internal-external rotation in association with taping. However, HDf for both KT and ST vs. NT was significantly higher in the ankle joint mediolateral direction only. These results suggest different sensorimotor feedforward and feedback mechanisms associated with retaining postural control.

## Data Availability

The datasets generated for this study are available on request to the corresponding author.

## Ethics Statement

All necessary information has been provided. The Charles Sturt University Human Ethics Committee approved the study, and all participants provided written consent having been informed about the research process and aims in accordance with the Declaration of Helsinki.

## Author Contributions

LH, LD, and JP collected the data. HJ, LD, LH, and JP performed the data analysis. HJ, KK, JP, AK, LH, and LD contributed equally to the interpretation of results and writing the paper, conceived and contributed substantially to the research design.

### Conflict of Interest Statement

The authors declare that the research was conducted in the absence of any commercial or financial relationships that could be construed as a potential conflict of interest.
